# Microfocused Ultrasound in Regenerative Aesthetics: A Narrative Review on Mechanisms of Action and Clinical Outcomes

**DOI:** 10.1111/jocd.16658

**Published:** 2024-11-05

**Authors:** Vasanop Vachiramon, Tatjana Pavicic, Gabriela Casabona, Jeremy B. Green, Jennifer Levine, Je‐Young Park, Julieta Spada, Mariana Muniz, John Akers, Matthew Jackson, Alec McCarthy

**Affiliations:** ^1^ Division of Dermatology, Faculty of Medicine Ramathibodi Hospital Mahidol University Bangkok Thailand; ^2^ Private Practice for Dermatology & Aesthetics Munich Germany; ^3^ Ocean Clinic Marbella Málaga Spain; ^4^ Skin Research Institute & Skin Associates of South Florida Coral Gables Florida USA; ^5^ Lenox Hill Hospital and Manhattan Eye, Ear, and Throat Hospital New York New York USA; ^6^ Apkoo‐Jung Oracle Dermatology Clinic Seoul Republic of Korea; ^7^ Julieta Spada Dermatology & Aesthetics Buenos Aires Argentina; ^8^ Dermatology Private Office São Paulo Brazil; ^9^ Global Medical Affairs Merz Aesthetics Raleigh North Carolina USA

**Keywords:** energy‐based devices, MFU‐V, microfocused ultrasound with visualization, regenerative aesthetics, Ultherapy

## Abstract

**Background:**

Microfocused ultrasound with visualization (MFU‐V) is widely used in aesthetic medicine for skin tightening and rejuvenation. However, its role in regenerative aesthetics and its precise mechanism of action are not fully understood.

**Objective:**

This narrative review aims to contextualize and articulate the mechanism of action of MFU‐V, evaluate its role in regenerative aesthetics, and assess its effectiveness based on existing clinical, histological, and skin‐mechanical studies.

**Methods:**

A comprehensive literature search was performed to collect and analyze studies on MFU's biological mechanisms, clinical outcomes, and impact on extracellular matrix (ECM) regeneration. The review integrates findings from clinical trials, histological analyses, and biomechanical assessments to provide a cohesive understanding of MFU‐V's role in aesthetic medicine.

**Results:**

MFU‐V emits focused ultrasound energy that penetrates multiple skin layers and the superficial musculoaponeurotic system, creating localized thermal coagulation points. These points initiate biological responses that recruit fibroblasts and stimulate the production of new collagen and elastin fibers. Enhanced ECM protein synthesis leads to significant improvements in skin biomechanics and quality, reducing skin laxity and enhancing appearance. Clinical studies support these findings, showing improvements in skin firmness and texture following MFU‐V treatment.

**Conclusion:**

Through analyzing the underlying biological mechanisms and the observable clinical outcomes, this narrative review sets the stage for a comprehensive understanding of the mechanism of action and role of MFU‐V in regenerative aesthetics.

## 1 Introduction

Regenerative aesthetics is a sub‐field of regenerative medicine focusing on returning skin and deeper tissues, such as the superficial musculoaponeurotic system (SMAS), to a more youthful structure and function by leveraging the body's regenerative pathways to address the aesthetic impact of aging [1, 2]. Since its introduction, various aesthetic treatments have self‐identified as regenerative aesthetic treatments, many articulating a unique approach and mechanism to achieving regeneration of tissues or their primary constituents. For example, stem cells or tissue fragments can modify their environment through cellular differentiation or via the recruitment and modulation of endogenous stem cells [1, 3]. In addition, biocues may stimulate cell signaling to improve the targeted tissue's microenvironment. Bio‐cue treatments may include platelet‐rich plasma (PRP) injections, exosomes, polymicronutrients, or growth factors or their stimulation with energy‐based devices (EBDs) [4, 5, 6]. Acellular regenerative scaffolds made of natural or synthetic materials such as calcium hydroxylapatite (CaHA; Radiesse) and poly‐l‐lactic acid (PLLA; Sculptra) leverage mechanoregulation or localized subclinical inflammation, respectively, to initiate signaling pathways that can affect cellular function down to the level of gene expression [7, 8, 9, 10, 11]. Chiefly, and despite having different mechanisms of action and treatment regimens, such treatments accomplish aesthetic correction by sufficiently regenerating components of target tissues to improve the overall tissue function, resulting in an aesthetic improvement. Since energy‐based devices (EBDs) achieve aesthetic corrections without implanted gels or biomaterials and the direct delivery of cells or biocues, it is reasonable to assume that aesthetic correction from such devices results from the body's physiological response to the treatment. The aim of this narrative review was to evaluate the evidence supporting EBDs, specifically microfocused ultrasound (MFU), in regenerative aesthetics.

## 2 Methods

A systematic literature search was conducted using established databases (PubMed, Scopus, and Web of Science) to identify relevant peer‐reviewed articles published up to July 2024. Search terms included combinations of “microfocused ultrasound,” “MFU‐V,” “Ultherapy,” “energy‐based devices,” “regenerative aesthetics,” “extracellular matrix,” “collagen,” “elastin,” “skin biomechanics,” and related terms. Studies investigating the cellular, histological, and functional changes in target tissues following MFU treatment for aesthetic purposes were included. Data extraction and analysis were performed by two independent reviewers, with discrepancies resolved through consensus. The findings were synthesized narratively, focusing on the mechanism of action, efficacy, and safety of MFU in the context of regenerative aesthetics.

## 3 Results and Discussion

### 3.1 EBDs in Regenerative Aesthetics

As regenerative aesthetics has matured, claims have surfaced involving regenerative mechanisms of action among devices with variable levels of evidence. Some EBDs with potential regenerative properties or with current regenerative science include lasers [12], radio frequency [13], microneedling, [14] microneedling with radiofrequency [15], and high‐intensity focused ultrasound (HIFU) [16]. Most energy‐based devices use heat or mechanical wounding to induce ECM protein synthesis, eventually resulting in a wide range of aesthetic outcomes ranging from tissue tightening to skin quality improvements. Applications of HIFU target the multilayer dermis and fibromuscular layers rich in fibroblasts to stimulate a regenerative effect [17]. Within the broad category of HIFU devices are microfocused ultrasound (MFU) devices. Key factors that differentiate MFU from HIFU are the consistency and spacing of the denatured tissue areas, the size and shape of the denatured tissue area, the consistency of the energy levels delivered to the tissues, and how these factors affect the surrounding tissue such as the epidermis and hypodermis/adipose tissue [18]. Ultherapy (Merz North America, Inc. Raleigh, N.C., USA) utilizes two ultrasound modalities—precise microfocused ultrasound energy delivery and real‐time visualization through collimated ultrasound or microfocused ultrasound with visualization (MFU‐V) [19]. Ultherapy is US‐FDA‐cleared to achieve lifting of the brow, lifting of lax submental and neck areas, improving lines and wrinkles on the décolleté, and visualization of the dermal and subdermal tissue layers. Other worldwide indications include noninvasive dermatological sculpting and lifting of the dermis in the upper face, lower face, neck, and décolleté, treatment of axillary hyperhidrosis, and sagging jawline lift.

### 3.2 Structural and Functional Components of the ECM

The ECM serves as a foundational and functional scaffold in regenerative aesthetics, influencing cellular behaviors through mechanisms encompassing growth factor modulation, cellular adhesion, cytokine activity, cellular migration, differentiation, growth, and cell death [20]. The structural complexity of the ECM is evidenced by its diverse components, notably collagens—particularly type I and type III in regenerative aesthetics—elastin, proteoglycans, glycosaminoglycans (GAGs), including hyaluronic acid, laminins, and fibronectin [21].

Collagen can be categorized into fibril‐forming collagens like type I and III or network‐forming collagens like the basement membrane collagen IV, providing structural support to the ECM. The ratios of collagen I to collagen III are important for gauging tissue strength and health [7, 22]. Elastin is an extensively crosslinked protein that provides elastic recoil and extensibility within the skin, among other tissues [23]. Collagen‐to‐elastin ratios are crucial in maintaining skin integrity, elasticity, and wound healing, with imbalances leading to aging and connective tissue disorders. Elastin also provides bio‐feedback to influence fibroblast activity and ECM remodeling [24]. Proteoglycans consist of the polysaccharide chains, GAG, bound to central proteins to construct a stabilizing ground substance in the ECM, providing hydration, resistance to external pressures, and aiding cell migration [25, 26]. Hyaluronic acid (HA) is a GAG date providing tissue hydration and osmotic balance among its many functions [27]. Laminin protein plays a role in proliferation, differentiation, migration, and adhesion within the ECM and can improve tissue regeneration after injuries [28]. Fibronectin, an ECM protein, forms fibrillar scaffolds for other ECM proteins, including collagens and proteoglycans. It supports maturation and attachment, cellular migration, and mechanosignalling and plays a major role in wound healing [29].

The complexity of the ECM is profound, as even a brief examination of its structure and function shows the array and interdependency of its various components. Regenerative aesthetics treatments that target different tissue planes of the skin and fibromuscular layer can affect the ECM to promote a healthy, youthful environment. These effects are externally observable and substantiated across multiple established aesthetic endpoints in various clinical trials, details of which will be covered in the subsequent sections.

### 3.3 The Role of Tissue Planes in ECM Regeneration

**FIGURE 1:**
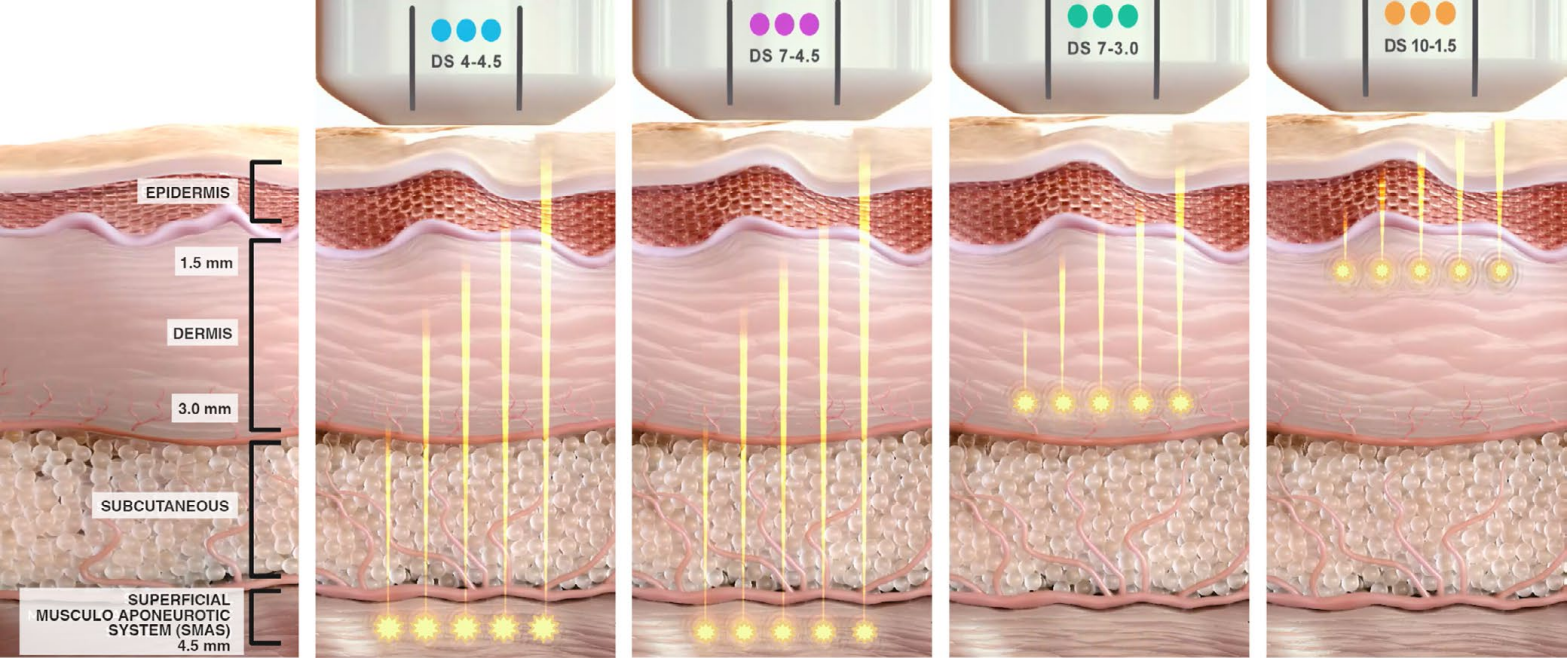
MFU‐V transducers targeting various skin layers (SMAS, reticular/papillary dermis) at specific depths (4.5 mm, 3.0 mm, 1.5 mm), demonstrating precise depth‐controlled thermal coagulation. DS, DeepSee.

Due to multiple factors, including age, sex, and body mass index (BMI), the skin thickness and anatomy of the skin varies between each unique patient [30, 31, 32, 33]. Patient assessment is vital to delivering personalized, quality care to each individual. The visualization component of MFU‐V allows providers to analyze the anatomy of the skin up to 8 mm below the surface, integral in targeting the optimal tissue layers, including the dermis, deep dermis, the fibromuscular layer partially consisting of the superficial musculoaponeurotic system (SMAS) and fibromuscular layer (Figure 1) [34]. Applying varying amounts of pressure based on the providers' discretion while maintaining proper coupling can increase this depth by up to 1.5 mm. Notably, MFU‐V's real‐time visualization and microfocused energy features allow for trained practitioners to bypass layers, including the epidermis, to minimize or prevent patient downtime and reduce the risk of post‐inflammatory hyperpigmentation (PIH) associated with resurfacing lasers, ultimately providing safe and effective single and repeat treatments [35]. Furthering personalized treatment, MFU‐V allows for further customization of treatment depths, treatment transducer (TD) width, and energy settings to address the specific needs of each unique patient for optimized lifting and tightening (Table 1). MFU‐V can target and treat deeper tissue planes than many other EBD and HIFU while maintaining similar patient comfort compared to more superficial aesthetic ultrasound [36]. Features of MFU‐V provide the tools to precisely treat the correct tissue planes, leading to an optimal response within the surrounding tissue and extracellular matrix (ECM) [37].

**TABLE 1:** Transducer setting and associated energy levels in Joules (J).

Transducer	Clinical application	Depth (mm)	Thermal coagulation point intensity				
			Level 4 (J)	Level 3 (J)	Level 2 (J)	Level 1 (J)	Level 0 (J)
DS 4‐4.5	Deep dermis and SMAS	4.5	1.20	1.00	0.90	0.75	0
DS 7‐4.5	SMAS	4.5	1.05	0.90	0.75	0.66	0
DS 7‐3.0	Dermis	3.0	0.45	0.35	0.30	0.25	0
DS 7‐3.0N	Dermis (narrow)	3.0	0.45	0.35	0.30	0.25	0
DS 10‐1.5	Superficial dermis	1.5	0.25	0.20	0.18	0.15	0
DS 10‐1.5N	Superficial dermis (narrow)	1.5	0.25	0.20	0.18	0.15	0

Abbreviation: DS, DeepSee.

## 4 MFU‐V Mechanism of Action

### 4.1 Introduction to the Dual Ultrasound MOA

**FIGURE 2:**
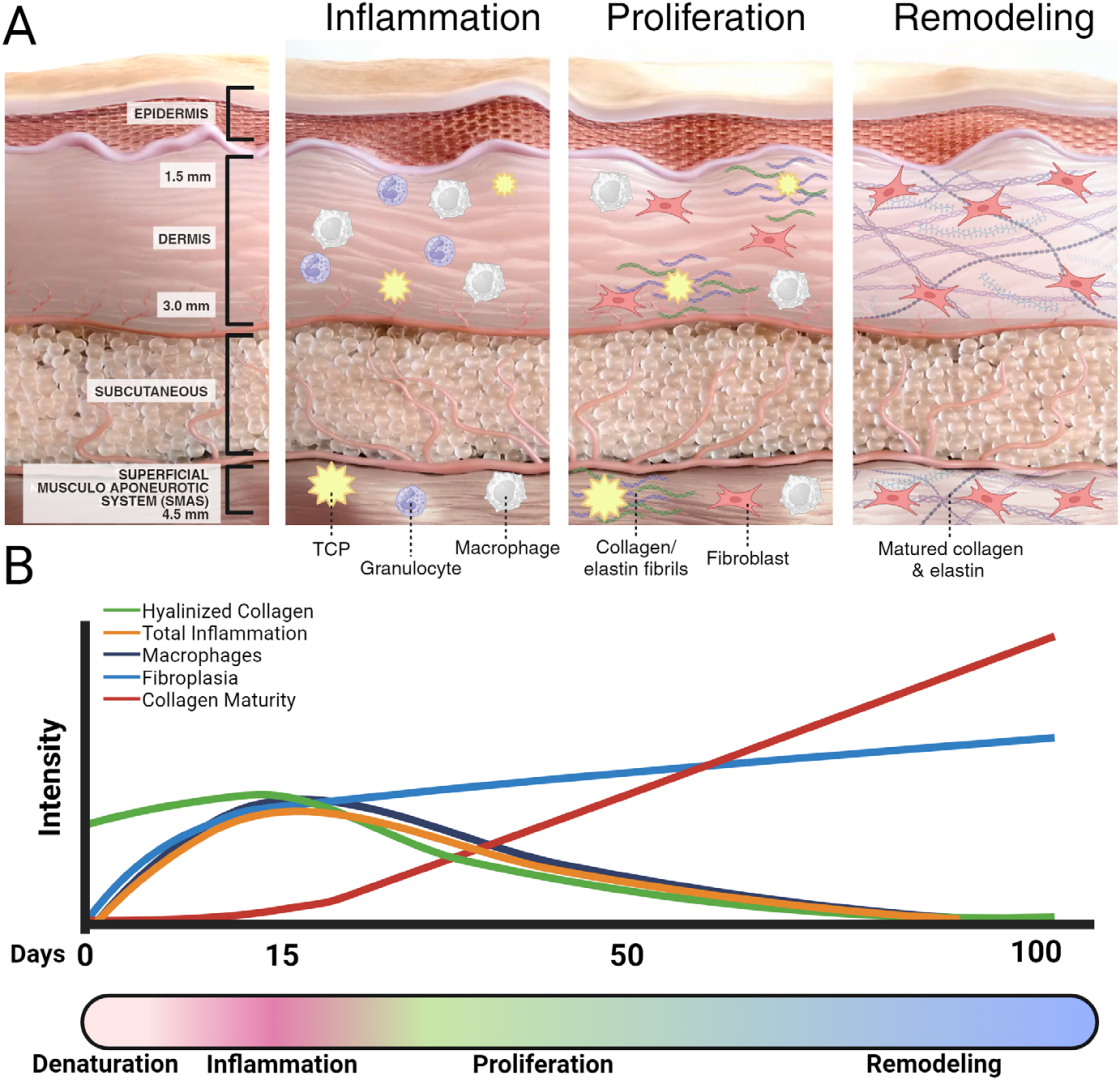
MFU‐V's mechanism of action for skin regeneration. (A) Three phases: Inflammation (TCP triggers immune response), Proliferation (fibroblasts produce new collagen/elastin), and Remodeling (collagen/elastin mature, restructuring skin). (B) Conceptual timeline and illustrative relative intensity of biological processes over 100 days. The *y*‐axis represents qualitative intensity.

MFU‐V's regenerative mechanism of action can be broken down into four steps. The first is the delivery of energy and precise denaturation of the local tissue. This initiates the body's natural healing process through the creation of thermal coagulation points (TCP), followed by temporary inflammation, cellular proliferation, and tissue remodeling, ultimately leading to significant increases in mature collagen and elastin (Figure 2) [38, 39]. Table 2 provides detailed information on studies exploring the regenerative potential of Microfocused Ultrasound (MFU).

**TABLE 2:** Overview of studies investigating the regenerative potential of microfocused ultrasound (MFU).

Study characteristics				Participants characteristics					Exposure/intervention			Outcomes
Author, year	Country	Design	Follow‐up period	No. participants	Sex (%F)	Health status/characteristics	Age	Ethnicity	Treated sites	Device (Brand)	Treatment	Categories
Denaturation												
Kist et al. 2006 [13]	n/s	In vivo study with 3 subjects	Immediate, 24 h, and 6 m	3	n/s	Healthy	n/s	n/s	Pre‐auricular region	Thermacool device	Multiple pass, low‐energy RF treatment (varying Joules and passes) versus single pass, high‐energy RF treatment	Multiple low‐energy passes (97 J) increased collagen damage similar to single high‐energy (166 J) pass, with 50% collagen replacement in 6 months, enhancing skin tightening
White et al. 2007 [40]	USA	In vivo study on cadaveric facial tissue	No follow‐up	6 cadavers	33% female (2/6)	n/a	49–72 years	Mixed	Superficial musculoaponeurotic system (SMAS)	Ulthera (Ultherapy), Intense Ultrasound System	Ultrasound therapy to create thermal injury zones in the SMAS layer	Intense ultrasound therapy created thermal injury zones up to 7.8 mm deep in the SMAS layer, with precise targeting and minimal surrounding tissue damage
Temporary immune response												
Marquardt et al. 2024 [38]	Germany, USA	Non‐clinical study on histological evolution of TCPs post‐MFU‐V treatment	14 and 90 days	n/a	n/a	Yucatan Miniature Swine models	n/s	n/a	Face and subcutaneous tissue (SMAS)	Ulthera (MFU‐V, Merz Aesthetics)	Microfocused ultrasound targeting depths of 1.5, 3.0, and 4.5 mm, TCP formation, collagen remodeling, and elastin neogenesis	Inflammation was mild and transient following treatment; Demonstrates elastin neogenesis and neocollagenesis after MFU‐V treatment; significant fibroblast recruitment to the TCP areas
Suh et al. 2015 [39]	South Korea	Prospective study with histologic evaluation	2 m	11	90.9% female (10/11)	Patients with facial skin laxity	35–64 years	Asian	Face and neck	Doublo (HIRONIC Co.)	Intense focused ultrasound (IFUS) for facial tightening; two handpieces with 3 and 4.5 mm focal depths	Increased collagen fibers without signs of inflammation or fat necrosis; increased fibrosis between fat layers confirmed histologically
Vachiramon et al. 2023 [43]	Thailand	Prospective, pilot study on combined HA filler and MFU treatments	56 days	13	92.3% female	Healthy subjects, HA filler recipients	Mean age 37.8 years	Fitzpatrick skin types III‐V	Abdomen (filler injection sites)	Ulthera (MFU), Belotero Balance HA filler (Merz Aesthetics)	MFU performed on the same day, day 14, and day 28 after HA filler injection, histological analysis of HA loss at different intervals	HA degradation observed when MFU performed within 14 days post‐HA injection; no significant histological changes after 28 days; no inflammatory reactions or granuloma
Casabona et al. 2014 [44]	Brazil	Clinical and histological study	180 days	1	100% female	Healthy subject undergoing inner thigh surgery	45 years	n/s	Inner thighs and retroauricular area	Ulthera (MFU‐V), Radiesse (CaHA)	MFU‐V combined with Radiesse and hyaluronic acid fillers	Neocollagenesis and neoelastogenesis after combined treatment; filler safety with MFU‐V
Keagle et al. 2001 [45]	USA	Animal study on linear rodent wound healing	1, 2, 5, 14, and 28 days	3 rodents	n/a	Healthy	n/a	n/a	Dermis and epidermis	n/a	Wounding followed by expression analysis of heat shock proteins	Heat shock proteins (HSPs) Hsp 47, 72, 32 expression in wound healing; collagen synthesis involvement
Hantash et al. 2009 [46]	USA	Prospective, histological study	10 weeks	n/s	n/s	Healthy	n/a	n/a	Dermis	Renesis System (bipolar fractional RF)	Bipolar fractional RF treatment	Induction of neocollagenesis and neoelastogenesis, dermal remodeling, collagen replacement
Ishida et al. 2011 [47]	Japan	Review article	n/a	n/a	n/a	n/a	n/a	n/a	n/a	n/a	Molecular chaperone HSP47 interaction with collagen	Collagen maturation, molecular chaperones
Proliferation												
Marquardt et al. 2024 [38]	Germany, USA	Non‐clinical study on histological evolution of TCPs post‐MFU‐V treatment	14 and 90 days	n/a	n/a	Yucatan Miniature Swine models	n/s	n/a	Face and subcutaneous tissue (SMAS)	Ulthera (MFU‐V, Merz Aesthetics)	Microfocused ultrasound targeting depths of 1.5, 3.0, and 4.5 mm, TCP formation, collagen remodeling, and elastin neogenesis	Inflammation was mild and transient following treatment; demonstrates elastin neogenesis and neocollagenesis after MFU‐V treatment; significant fibroblast recruitment to the TCP areas
ECM rejuvenation via maturation/remodeling												
White et al. 2007 [40]	USA	In vivo study on cadaveric facial tissue	No follow‐up	6 cadavers	33% female (2/6)	n/a	49–72 years	Mixed	Superficial musculoaponeurotic system (SMAS)	Ulthera (Ultherapy), Intense Ultrasound System	Ultrasound therapy to create thermal injury zones in the SMAS layer	Selective thermal injury targeting SMAS for noninvasive facial rejuvenation, thermal collagen denaturation
Marquardt et al. 2024 [38]	Germany, USA	Non‐clinical study on histological evolution of TCPs post‐MFU‐V treatment	14 and 90 days	n/a	n/a	Yucatan Miniature Swine models	n/s	n/a	Face and subcutaneous tissue (SMAS)	Ulthera (MFU‐V, Merz Aesthetics)	Microfocused ultrasound targeting depths of 1.5, 3.0, and 4.5 mm, TCP formation, collagen remodeling, and elastin neogenesis	Inflammation was mild and transient following treatment; demonstrates elastin neogenesis and neocollagenesis after MFU‐V treatment; significant fibroblast recruitment to the TCP areas
Suh et al. 2015 [39]	South Korea	Prospective study, histologic evaluation	2 m	11	90.9% female	Facial laxity	35–64 years	Asian	Face and neck	Doublo (HIRONIC Co.)	Intense focused ultrasound with 3.0 and 4.5 mm transducers, multiple passes per treated area	Increased collagen fibers without signs of inflammation or fat necrosis; increased fibrosis between fat layers confirmed histologically
Laubach et al. 2008 [48]	USA	In vitro study on postmortem skin samples	n/a	n/a	n/a	Postmortem skin	n/a	n/s	Dermis	Ulthera Inc. prototype device	Intense focused ultrasound (IFUS) for precise thermal coagulation	Thermal damage, collagen denaturation
Gliklich et al. 2007 [49]	USA	Open‐label, phase 1 study	Immediate (within 24 h) and delayed (4–12 weeks)	15	60%	Patients scheduled for rhytidectomy	Mean 53 years (SD ± 7)	n/s	Face, neck	Prototype device (Ulthera Inc.)	Intense ultrasound therapy to deep dermal facial skin and subcutaneous tissues	Evaluation of clinical safety, histologic features, pain, and inflammation
Suh et al. 2011 [50]	South Korea	Prospective study, histologic analysis	2 m	22	90.9% female	Facial laxity	Mean age 48.5 years	Fitzpatrick types III–VI	Nasolabial fold, jawline	Ulthera (IFUS)	Intense focused ultrasound for facial tightening, histologic evaluation of collagen production	Improvement in nasolabial folds and jawline laxity; increased dermal collagen and straighter elastic fibers in reticular dermis
Suh et al. 2019 [51]	South Korea	Prospective study, histologic analysis	2–3 m	10	100% female	Periorbital wrinkles	45–73 years	Fitzpatrick types III‐IV	Periorbital (crow's feet)	Ulthera System (Ulthera Inc.)	Intense focused ultrasound using 1.5 mm transducer, targeting fine and deep wrinkles in periorbital area	Moderate to good improvement in periorbital wrinkles; increased collagen and elastic fiber density, minimal side effects (welts, erythema)
Yutskovskaya et al. 2020 [52]	Russia	Randomized, split‐face comparative clinical study	15 m	20	100%	Age‐related skin laxity	35–45 years	n/s	Lower face, neck, décolleté, abdomen	Ulthera (MFU‐V) + Radiesse (CaHA diluted)	Combination of diluted CaHA and MFU‐V; increased collagen and elastin fibers, enhanced neocollagenesis, and skin remodeling	Improvement in age‐related changes, high patient satisfaction; histological evaluation of collagen I/III, Ki67, angiogenesis
Suh et al. 2012 [53]	South Korea	Prospective, clinical study on infraorbital laxity treatment	6 m	15	86.67% female	Infraorbital laxity	27–69 years	Fitzpatrick types III–V	Lower eyelid (infraorbital laxity)	Ulthera System (Ulthera Inc.)	Intense‐focused ultrasound for infraorbital laxity, applied with a 7.0 MHz, 3.0 mm focal depth transducer	Improved infraorbital laxity, increased collagen and elastic fiber density; minimal side effects (erythema, edema, purpura)
Lin 2020 [54]	Australia	Prospective, clinical study on postpartum lower abdominal laxity	3 and 6 m	21	100% female	Postpartum lower abdominal skin laxity	25–40 years	Mostly Asian, some Caucasian	Lower abdomen (postpartum skin laxity)	Ulthera System (Ulthera Inc.)	MFU‐V using 1.5‐, 3.0‐, and 4.5‐mm transducers, 1140 lines total across lower abdomen	Significant improvement in skin laxity, increased collagen and fibrous septae thickness, no significant adverse events, high patient satisfaction at 6 months
Vachiramon et al. 2020 [55]	Thailand	Randomized, prospective, comparative study on abdominal skin laxity	1, 3, and 6 m post‐treatment	30 (28 completed)	100% female	Abdominal skin laxity	Mean age 43.3 years	Fitzpatrick skin type III	Abdomen (single‐ and dual‐plane)	Ulthera System (Ulthera, Inc.)	MFU‐V with 4.5 and 3.0 mm transducers (single and dual‐plane treatment for abdominal skin laxity)	Significant reduction in waist circumference for childbirth patients, comparable improvement for both protocols, pain scores recorded, transient erythema, and edema
Meyer et al. 2021 [56]	Brazil, USA, Chile	Experimental study on the effects of MFU on facial rejuvenation	45 and 90 days post‐treatment	30	100% female	Patients with facial skin aging (tissue laxity, wrinkles)	30–60 years	Mixed	Full face	Heros HIFU (Fismatek)	Single MFU session using 1.5, 3, and 4.5 mm transducers, energy ranging from 0.1 to 2.0 J	Clinical improvement in facial symmetry, increased collagen type I, improved firmness and wrinkle reduction; transient hyperemia and pain during treatment
Sasaki et al. 2021 [57]	USA	Prospective, single‐center, single‐blinded, nonrandomized study on collagen synthesis post‐MFU‐V	6 weeks	2	100% female	Healthy subjects, scheduled for rhytidectomy	30–65 years	n/s	Pre‐auricular region (face)	Ultherapy (MFU‐V; Merz North America, Inc.)	Dual density MFU‐V (30 lines using 7–3.0 mm transducer, 30 lines using 4–4.5 mm transducer)	Increased Type I and Type III collagen synthesis (26% and 60% increases, respectively); no adverse events; heavy water method to measure in vivo collagen synthes
Casabona et al. 2023 [58]	Spain	Case study of a combined collagen stimulation procedure	6 m	1	100% female	Healthy 60‐year‐old patient undergoing facelift surgery	60 years	n/s	Face (pre‐auricular areas)	Ultherapy (MFU‐V), Radiesse (Ca‐HA filler), Belotero Revive (HA filler), Dermapen microneedling	MFU‐V combined with calcium hydroxylapatite‐based filler and hyaluronic acid filler, along with microneedling	Increased dermal and epidermal thickness, most effective in combined treatments; collagen organization improved; enhanced neocollagenesis in SMAS and retinacula cutis
MFU‐V mechanism of action												
Vachiramon et al. 2023 [43]	Thailand	Prospective, pilot study on combined HA filler and MFU treatments	56 days	13	92.3% female	Healthy subjects, HA filler recipients	Mean age 37.8 years	Fitzpatrick skin types III–V	Abdomen (filler injection sites)	Ulthera (MFU), Belotero Balance HA filler (Merz Aesthetics)	MFU performed on the same day, day 14, and day 28 after HA filler injection, histological analysis of HA loss at different intervals	HA degradation observed when MFU performed within 14 days post‐HA injection; no significant histological changes after 28 days; no inflammatory reactions or granuloma
Marquardt et al. 2024 [38]	Germany, USA	Non‐clinical study on histological evolution of TCPs post‐MFU‐V treatment	14 and 90 days	n/a	n/a	Yucatan Miniature Swine models	n/s	n/a	Face and subcutaneous tissue (SMAS)	Ulthera (MFU‐V, Merz Aesthetics)	Microfocused ultrasound targeting depths of 1.5, 3.0, and 4.5 mm, TCP formation, collagen remodeling, and elastin neogenesis	Inflammation was mild and transient following treatment; demonstrates elastin neogenesis and neocollagenesis after MFU‐V treatment; significant fibroblast recruitment to the TCP areas
White et al. 2007 [40]	USA	In vivo study on cadaveric facial tissue	No follow‐up (cadaveric study)	6 cadavers	33% female (2/6)	n/a (cadaveric)	49–72 years	Mixed	Superficial musculoaponeurotic system (SMAS)	Ulthera (Ultherapy), Intense Ultrasound System	Ultrasound therapy to create thermal injury zones in the SMAS layer	Selective thermal injury targeting SMAS for noninvasive facial rejuvenation, thermal collagen denaturation

Abbreviations: n/a, not applicable; n/s, not stated.

### 4.2 Denaturation

**FIGURE 3:**
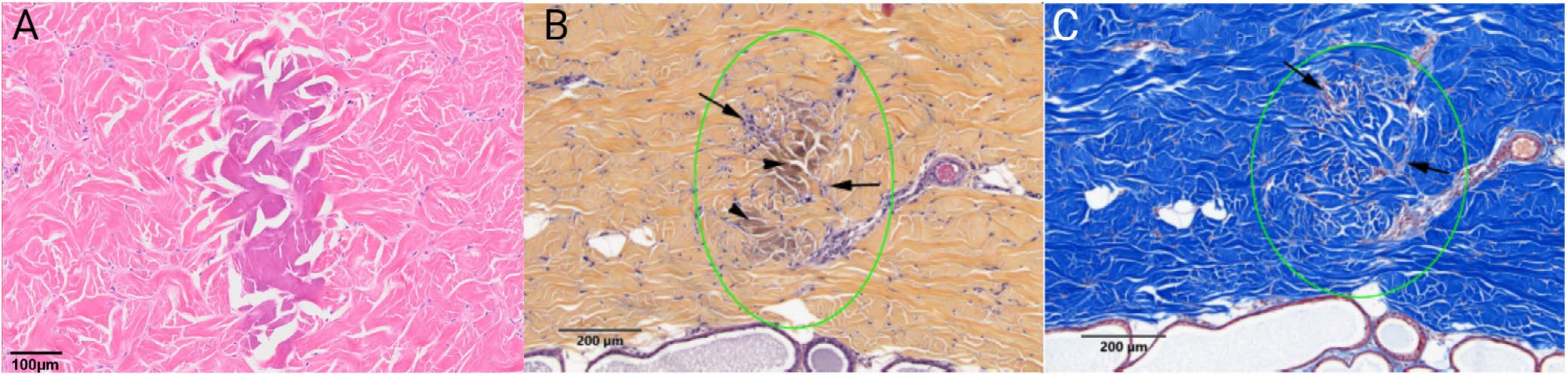
(A) Hematoxylin & Eosin (H&E) staining shows the inverted cone appearance of thermal coagulation points (TCPs) 14 days post‐treatment with an R&D transducer using 0.25 J at a 1 mm depth with 10 MHz. (B) Safranin‐Hematoxylin–Eosin (SHE) staining reveals collagen degradation (darker arrowheads) and early fibroplasia (arrows) 14 days after treatment with ~0.8 J at a 3.0 mm depth. (C) Masson's Trichrome (MT) staining highlights TCPs and hyalinized collagen within and around the TCP (green circle) 14 days post‐treatment with ~0.8 J at a 3.0 mm depth. Photos courtesy of Merz Aesthetics Skin Lab.

Collimated ultrasound visualization is utilized in real‐time to identify appropriate treatment planes, where precise microfocused ultrasound waves are delivered. These waves create vibrations within targeted tissue molecules, raising temperatures that induce collagen fibril denaturation and contraction, beginning with the disruption of intramolecular hydrogen bonds at approximately 57°C–58°C. This leads to immediate collagen contraction, as demonstrated in a cadaver study by White et al. [40]. Complete denaturation occurs at 65°C. The arrangement of TCPs, approximately 1 mm^3^ in size and shaped like inverted cones, in surrounding healthy tissue is critical for triggering a temporary immune response and promoting efficient healing (Figure 3). This process allows the body to attract cells that infiltrate and remodel the treated area. By assessing a patient's unique anatomical features with the collimated ultrasound, providers can optimize the delivery of microfocused ultrasound to these critical tissue planes while also visualizing and avoiding structures that should not be treated, such as vessels, nerves, and bone. The ultrasound treatment bypasses the skin's surface to deliver energy directly to collagen‐rich dermal and fibromuscular layers. This denaturing of proteins and other components within these targeted layers initiates the intrinsic collagen and elastin regeneration processes. The MFU‐V process is marked by TCPs, which are discrete, consistent in size, and optimally spaced when compared to those produced by other HIFU devices [13].

### 4.3 Temporary Immune Response

The inflammatory portion of the body's healing response is initiated by damage‐associated molecular patterns (DAMPs) that are released by affected tissue. These signals activate immune cells by binding pattern recognition receptors to stimulate temporary inflammatory pathways. This is followed by the release of cytokines and chemokines, which attract numerous cell types, including macrophages, involved in the healing process [41, 42]. In a 2024 animal study, skin tissue around TCPs was excised following the application of energy levels 2 and 4 at depths of 1.5, 3.0‐, and 4.5‐mm [38]. Histological analysis indicated the TCPs consisted of collagen, which exhibited a loss of its fine fibrillar structure, indicative of denaturation. From Day 14 to Day 90, there were observable changes in TCP size, cellular activity, and collagen maturity. Although the presence of macrophages and giant cells were noted, no inflammation was seen at 90 days. These findings were supported by a clinical trial that collected 11 tissue samples from patients after a single MFU‐V treatment at the 4.5 mm depth and 4.4 MHz frequency, revealing no epidermal changes or inflammatory reactions at 2 months [39]. Additionally, Vachiramon et al. found no inflammation at Day 56 in a clinical trial of 14 subjects studying the treatment combination of MFU‐V and hyaluronic acid (CPM‐HA 22.5 mg/mL, Belotero Balance, Merz Aesthetics, Raleigh, NC, USA) [43].

Animal tissue analyzed through H&E staining showed cells, including fibroblasts, macrophages, T‐cells, and a small number of giant cells, infiltrating and accumulating around the edges of the TCP at 14 days. By 90 days, these cells had significantly infiltrated the TCP [38].

### 4.4 Proliferation

The healing process requires a balance of protein degradation and synthesis. Throughout the proliferative phase of the healing process, matrix metalloproteinases (MMPs) expressed by fibroblasts and macrophages degrade denatured proteins to be replaced with granulation tissue consisting of immature collagen, fibronectin, and proteoglycans. Granulation tissue forms a scaffold for the cells involved in the healing process to migrate and differentiate, promoting mature tissue deposition [41]. Heat shock proteins (HSPs) also play a role during collagen and elastin regeneration in response to EBD treatment [44, 45, 46]. The collagen chaperone, Hsp47, assists in collagen replacement at TCP sites [47]. Marquardt et al. found a higher number of Hsp47‐positive cells in the tissue surrounding the TCP at 14 days, with 85.0% of cells being Hsp47‐positive but remaining on the border of the TCP. At 90 days, 98.9% of the cells were Hsp47 positive and in significantly higher concentrations within the TCP than in the surrounding tissue indicating infiltration leading to proliferation [38].

### 4.5 ECM Rejuvenation via Remodeling

Maturation, or remodeling, is the final step in the healing process. Mature elastin and collagen are formed, and collagen type III is converted into collagen type I. The creation of TCPs via targeted application of MFU‐V energy causes localized protein denaturing at the dermal planes [40, 48, 49]. This induces protein regeneration and ECM restructuring via healing processes to resemble a youthful‐associated ECM environment. Clinical studies utilizing qualitative and quantitative measures of skin quality and physiological function lend scientific support to the reasoning that dermal restructuring occurs through the regeneration and reorganization of proteins and key components. Studies of the face, neck, and lower abdomen have shown structural ECM recovery at the microanatomical level [50, 51, 52].

Histological analysis from Suh et al. on skin biopsies taken from the lateral cheek of 11 MFU‐V‐treated female patients before treatment and 2 months prior showed a 23.7% (*p* < 0.001) increase from baseline in collagen at the reticular dermis, with dermal mean thickness increasing from 1.32 to 1.63 mm (+65.9%, *p* < 0.001) supporting neocollagenesis. Elastin fibers were found straighter and more parallel following treatment, suggesting MFU‐V‐induced reorganization to a more youthful state [50]. Performing a similar histological analysis of biopsies from 11 patient cheeks 2 months after MFU non‐visual treatment with a specified 4 MHz, 4.5 mm probe at an energy level of 1.2 J, Suh et al. visualized increased collagen density of the reticular dermis [39]. In a 2019 study of a single MFU‐V treatment to the periorbital region with a 19 MHz, 1.5 mm probe at an energy level of 0.15–0.25 J, Suh et al. obtained three patients before‐and‐after biopsies at 2 to 3 months post‐treatment. While the histometric assessment was non‐significant, collagen density increased by 28.22% in the upper papillary dermis and 14.95% in the lower reticular dermis. The elastic fiber was reported to increase by 23.59% and 33.04% in the papillary and reticular dermis layers, respectively [51]. From Suh et al., two subjects receiving a single treatment of MFU‐V had punch biopsies from their lower eyelids, with significant loss of collagen and elastic fibers before treatment and showing regeneration of collagen and elastic fibers 6 months post‐treatment [53]. In addition, the elastin‐positive area in the TCPs of swine tissue at 14 days, compared to 90 days, was strongly increased [38].

In a study treating lower abdomen laxity postpartum, Lin applied MFU‐V within 6 months of childbirth using three standard transducers with variable frequency and focal depth to target specific anatomical layers of the anterior abdominal wall [54]. A single patient sample recovered from lower abdominoplasty 6 weeks post‐MFU‐V treatment showed a significant increase in total collagen in both the dermis and subcutaneous tissue of pretreated samples compared to controls with standard microscopy at 10× magnification, with further examination at 40× revealing denser and larger collagen bundles. Additionally, deeper analysis demonstrated an increase in the number and thickness of fibrous septae within the adipose layer between the dermis and Scarpa's fascia. However, adipocyte sizes in superficial and deeper fat compartments remained unchanged. These septae are thought to contribute to the smoothness/tautness of the skin surface. Despite a short interval and limited sample size, Lin and Suh et al. targeted the fascia and connecting fibrous septae at the SMAS and appeared to contribute to improved skin quality with MFU treatment. A similar finding was demonstrated by Vachiramon et al., with both single‐ (4.5 mm) and dual‐plane (4.5 and 3.0 mm) having similar effectiveness in treating abdominal skin laxity, particularly in patients who had undergone childbirth [55]. In addition to neocollagenesis and protein fiber reorganization, Meyer et al. demonstrated significant changes within a three‐patient histological sample collected 45 days following a single MFU treatment across facial regions. A substantial rise in collagen type I compared to type III was seen, along with significant increases in fibroblasts (*p* = 0.02) blood vessels (*p* = 0.0062), suggesting angiogenesis in dermal remodeling and inflammatory cells (*p* = 0.0036). Immunohistochemistry confirmed the presence of the CD68 macrophage marker, IHQ, indicating phagocytosis of necrotic adipose tissue and matched with fibrosis associated with increased fibroblast activity and collagen synthesis [56]. Sasaki et al. conducted a case series of two subjects involving a split‐face MFU‐V treatment on one side of the face in the pre‐auricular region and no treatment on the other side as the control [57]. The treatment included a triple‐depth, high density, 30 lines of treatment per transducer involving the following transducers and energy levels/frequency: 4 MHz, 4.5 mm (0.90 J), 7 MHz, 3.0 mm (0.30 J), and 10 MHz, 1.5 mm (0.18 J). At 1 month, collagen synthesis increased 8.4% for Collagen I and 16.8% for Collagen III compared to the tissue on the control side. Collagen I synthesis increased by 26% on average, and Collagen III increased by 60% on the treated side of the two subjects' faces compared to baseline. In a single patient case study undergoing sub‐SMAS facelift surgery, histological analysis from Casabona et al. on a tissue sample of monotherapy MFU‐V saw a modest improvement in SMAS thickness with a slight improvement in skin thickness overall and parallel organization of collagen fibers [58].

The emerging research surrounding MFU and MFU‐V treatments highlights a promising advancement in dermatological therapy by focusing on the rejuvenation and structural refinement of the dermal ECM. Studies have consistently demonstrated that targeted MFU‐V energy can effectively induce a regenerative process in the skin, leading to the restructuring of critical ECM components, primarily collagen and elastin. This enhances the skin's mechanical properties and functional integrity and replicates a more youthful ECM architecture. The histological evidence from various studies supports the efficacy of MFU‐V in promoting neocollagenesis, reorganizing elastin fibers, and increasing skin turgidity and thickness. These findings underline the potential of MFU‐V as a noninvasive option for skin rejuvenation, offering significant improvements in skin quality and structure, thereby contributing to the broader goals of aesthetic and therapeutic dermatology.

## 5 Improved Skin Physiological Function

In regenerative aesthetics, it is crucial to recover structural ECM proteins and quantify improved skin function. For instance, “regenerative” treatments should stimulate enough collagen to noticeably enhance skin firmness [1]. Various functional aspects of skin are pertinent in aesthetics, each linked to specific ECM proteins. These include but are not limited to skin firmness (collagen), elasticity (elastin), hydration (proteoglycans), and oxygenation (angiogenesis) [10].

A noticeable manifestation of physical aging includes changes in skin biomechanics. When cells age and ECM protein production slows, the mechanical implications include decreased firmness and elasticity, which contribute to increasing wrinkle severity and skin laxity [59, 60]. Thus, validating regenerative aesthetic treatments requires measuring structural regeneration and skin mechanics. The Cutometer is a widely used, clinically validated, noninvasive tool to measure skin biomechanics. It applies suction to the skin and measures its resistance to deformation (firmness) and elastic recoil (elasticity) [61, 62]. In Cutometer measurements, R2 and R5 assess skin elasticity. R2, known as “gross elasticity,” measures the skin's overall elasticity [63]. On the other hand, R5, known as “net elasticity,” also measures the skin's elastic recovery after being stretched. Using cutometry, Kerscher et al. conducted a 22‐patient study that evaluated the short and long‐term impact of a single MFU‐V targeting preselected depths of 4.5 and 3.0 mm on skin biomechanics [64]. Short‐term results indicated no significant reductions in skin temperature, erythema, hydration, or barrier function. The results showed a significant decrease in R2 and R5 values 4 weeks post‐treatment, suggesting a physiological restructuring of collagen tissue. However, both elasticity parameters significantly increased at 12‐ and 24‐week post‐treatment, indicating an improvement in skin firmness. This pattern of initial reduction followed by improvement in elasticity aligns with the expected outcomes of collagen and elastin remodeling induced by MFU‐V treatment [64].

A 22‐patient split‐face study by Lee et al. evaluated the differences in pore size and skin biomechanics following a single treatment using the 10‐MHz 1.5 mm on one side of the face and the 7‐MHz 3.0 mm transducer on the other. When assessing R2 (gross elasticity), R5 (net elasticity), and R7 (biological elasticity) using a Cutometer, it was found that within the first 3 weeks, R2, R5, and R7 were most improved with a 1.5 mm transducer. However, 6 months post‐treatment, skin treated with a 3.0 mm transducer had significantly higher elastic values across all three R measures. This suggests that a multiple, deeper‐depth treatment holds advantages over a single‐depth superficial treatment in restoring skin elasticity [65]. These results suggest that an initial thermal degradation of collagen is followed by collagen and elastin synthesis, resulting in improved skin firmness and elasticity relative to baseline, highlighting the advantages of targeting deeper skin layers to improve the biomechanical properties of the skin.

In addition to modulating skin biomechanics, another important hallmark of youthful skin is the presence of oxygen [10]. Oxygen is crucial for the physiological function of skin cells and tissues and exerts anti‐inflammatory and anti‐hypoxic effects on skin tissues [66]. In aesthetics, other regenerative treatments have been shown to increase markers of angiogenesis, improve vascular density and perfusion, increase skin hemoglobin content, and improve skin radiance [6, 52, 67, 68]. A study by Araco evaluated the hemoglobin content in a cohort of patients treated with the 1.0, 3.0, and 4.5 mm transducers in a single session. Hemoglobin content was measured with Antera 3D and revealed that 12 months post‐treatment, a 39.3% increase in hemoglobin content was achieved [68].

## 6 MFU in Regenerative Combination Treatments

Combining MFU‐V with injectable treatments like CaHA, PLLA, and HA fillers is an emerging strategy in regenerative aesthetics. This approach leverages the ECM regenerating properties of MFU‐V alongside the volumizing and biostimulatory effects of various fillers and regenerative biostimulators. Such a combination has gained clinical favorability, as it targets multiple aspects of skin aging, including laxity and volume loss, in a single treatment protocol. Combination treatments utilizing MFU‐V and HA fillers may enhance the overall visual outcomes of treatments, as the mechanical support from fillers potentially complements the tissue tightening induced by MFU‐V. Similarly, combination with regenerative biostimulators may multiply the biostimulatory effect on ECM proteins. In addition, MFU‐V is occasionally deployed alongside other EBDs or with multiple adjunctive therapies.

The combination of MFU‐V and CaHA fillers, particularly CaHA, is the most widely reported combination treatment, with at least 18 publications demonstrating their combined use and efficacy. Central to the rationale of combining CaHA and MFU‐V is the understanding that both modalities have different mechanisms of action that may function synergistically. Additionally, as with other fillers, undiluted or minimally diluted CaHA can create volume, while MFU‐V can tighten the skin around the added volume, enhancing its perceived effect. Studies combining diluted or hyperdiluted CaHA would rely on a synergistic regenerative mechanism of action, as diluting CaHA beyond a 1:1 dilution ratio minimizes the direct volumizing effect [69]. One study by Casabona et al. examined the histological results of combined treatments deployed at different time intervals, observing that MFU‐V followed immediately by CaHA injections yielded the most efficacious results [58]. It is hypothesized that treating EBDs first may yield enhanced results, as the mechanism of action, TCP induction, may damage new collagen created as a foundation from the CaHA injections. Yutskovskaya et al. evaluated the combination of (MFU‐V) and CaHA diluted 1:2 with normal saline in 20 subjects. The results showed significant improvement in age‐related changes, with marionette line scores improving from 2.47 ± 0.8 to 1.8 ± 0.7 (*p* ≤ 0.00003), jawline contour scores from 2.2 ± 0.7 to 1.89 ± 0.56 (*p* ≤ 0.005), and neck scores from 2.1 ± 0.7 to 1.7 ± 0.6 (*p* ≤ 0.005) after 15 months, alongside high patient satisfaction and minimal adverse effects [52].

In addition to combinations with CaHA, several studies have explored combinations of MFU‐V with polymer injections. PLLA is a thermoplastic biopolymer that, when injected into the skin, can restore several proteins in the ECM, leading to gradual volumization [70]. Of the three studies combining MFU‐V and PLLA, none investigated histological, biomechanical, or volumetric changes but rather made clinical suggestions and showcased favorable outcomes with before‐and‐after photographs [71, 72, 73]. Jerdan and Fabi postulate on the clinical synergy of PLLA and MFU‐V by suggesting concurrent, multi‐plane induction of ECM protein stimulation [73]. In addition to PLLA, a single study has reported the combination of MFU‐V and polymethylmethacrylate‐collagen filler (PMMA; Bellafill, Suneva Medical, San Diego, CA). PMMA is a permanent filler that has been shown to drive neocollagenesis. This study conducted post‐treatment histology to examine evidence of energy treatment, inflammation, presence of PMMA, and intactness of the PMMA. Samples treated with both PMMA and MFU‐V show that PMMA particles were present, intact, accompanied by inflammation, and had histological evidence of the energy treatments. This study concluded that the lymphohistiocytic effect was mostly attributed to the PMMA microspheres and that energy delivered from the MFU‐V was sufficiently low to preserve their structure and stability in situ [74].

Several studies have also evaluated the combination treatment of MFU‐V with HA dermal fillers. These studies have shown that combining MFU‐V with HA fillers yields improvements in volumization and pore size reduction [75]. Like studies combining MFU‐V and CaHA or PLLA, the treatment order must be considered, as treating areas previously filled with HA filler results in filler volume loss and network integrity compromise [43]. Despite the accidental degradation of HA gels, such treatments are safe and do not hinder the collagen‐synthesizing effect of MFU‐V [44]. Noting these observations, it is suggested that MFU‐V before dermal filler may yield the most effective outcomes. In cases where volumization or superficial line treatment may augment the skin tightening effect of MFU‐V, such combination treatments may be deployed.

In addition to fillers, MFU‐V has been evaluated with many other aesthetic treatments, including botulinumtoxinA, ascorbic acid, lasers, and other EBDs [72, 76, 77, 78, 79, 80, 81]. Most studies evaluating unique combinatorial uses highlight their safety and efficacy. A list of the study types, clinical targets, and combination therapies used are given in Table 3.

**TABLE 3:** Combination uses of MFU‐V and other aesthetic procedures.

Reference	Study type	Pathology treated	Adjunctive therapy	Location	Age, sex, ethnicity	Outcome
Aksenenko, 2019 [77]	Controlled, comparative study	Facial aging	Fractional CO_2_ laser	Russia	47–55 years, 100% female, n/s	Skin density increased by 22.5% (combined group) versus 13% (control); recovery time reduced from 22 to 10 days
Barbarino, 2021 [87]	Case series	Tear trough deformities	HA filler (Belotero balance)	USA, Netherlands, Australia	35–65 years, 100% female, n/s	Physicians rated 90% “very much improved” after combined treatment versus 0% initially; all 10 participants very satisfied
Bartsch et al., 2020 [88]	Randomized interventional prospective study	Skin laxity & dimpling	CaHA filler (Radiesse), tissue stabilized guided subcision (Cellfina)	Austria, Spain, USA, Germany, Philippines	37.2 ± 6.8 years, 100% female, n/s	Showed the highest improvement in skin laxity (2.23 odds) and dimpling (1.79 odds)
Bozkurt & Tatar, 2021 [89]	Case report	Burn scars	Fractional CO_2_ laser, nanofat injections	Turkey	18 years, 100% female, n/s	Reduced burn scar hardness, swelling, and itching in two sessions
Carruthers et al., 2016 [90]	Consensus	Aesthetic correction	HA (Belotero) and CaHA (Radiesse) fillers	USA, Canada	n/s, mixed, n/s	Expert consensus recommends using MFU‐V before injectable agents like BoNT and fillers for optimal facial rejuvenation
Casabona & Marchese, 2017 [91]	Objective, nonrandomized study	Stretch marks	Dilute CaHA (Radiesse), microneedling, and ascorbic acid	Brazil	21–34 years, 100% female, n/s	Significantly improved stretch marks, decreasing scar scores by 4.9 points.
Casabona & Michalany, 2014 [44]	Histological	Aesthetic collagen loss	HA filler (Juvederm Voluma), CaHA filler (Radiesse)	Brazil	45 years, 100% female, n/s	Enhanced collagen and elastin production without causing granulomas or altering filler properties
Casabona & Pereira, 2017 [92]	Retrospective; histological	Skin laxity and cellulite	CaHA filler (Radiesse)	Brazil	18–55 years, 100% female, n/s	Significantly improved cellulite severity in 90 days, with a 4.5‐point CSS improvement
Casabona & Teixeira, 2018 [93]	Retrospective	Skin laxity and lines of the neck & décolletage	CaHA filler (Radiesse)	Brazil	35–55 years, 100% female, n/s	Improved neck and décolletage lines by at least one grade in 90 days
Casabona et al., 2023 [58]	Histological	Aesthetic collagen loss	HA filler (Belotero), CaHA filler (Radiesse)	Spain, USA, Serbia, Germany	60 years, 100% female, n/s	Tripled epidermal and dermal thickness, showing a synergistic effect in collagen production
Casabona, 2018 [78]	Prospective pilot study	Atrophic acne scars	CaHA filler (Radiesse)	Brazil	35–55 years, 100% female, not specified	Significantly improved atrophic acne scars, reducing severity scores by 50% in 90 days
Casabona, 2019 [94]	Objective, nonrandomized study	Stretch marks	CaHA filler (Radiesse), microneedling, and ascorbic acid	Spain	18–55 years, 100% female, n/s	Reduced Manchester Scar Scale scores by 33%, achieving high satisfaction
Casabona, 2022 [95]	Case series	Skin laxity of the chest & buttocks	CaHA filler (Radiesse)	Spain	38–60 years, 75% female, n/s	Improved chest and buttock skin laxity by 2 grades after 2–3 treatment sessions, with customized protocols
Chao et al., 2017 [96]	Consensus	Aesthetic correction	HA fillers, BONT‐A, CaHA filler	Taiwan, India, Australia, USA, Germany, Hong Kong, Thailand	n/a, n/a, Asian	Pan‐Asian consensus recommends combining MFU‐V with fillers and BoNT‐A for customizing facial enhancements, focusing on oval shape
Coleman & Pozner, 2016 [97]	Case series	Skin laxity and cellulite	Subcision, fat transplantation	USA	n/s, not specified, n/s	Combining MFU‐V with other treatments for thigh and buttock rejuvenation enhances outcomes by targeting multiple layers safely
Fabi et al., 2016 [98]	Consensus	Aesthetic correction	BONT‐A, HA filler, CaHA filler (Radiesse)	USA	n/a, n/a, all Fitzpatrick skin types	Consensus recommends combining MFU‐V with fillers and BoNT for optimal rejuvenation of the neck, décolletage, and hands
Fabi et al., 2016 [99]	Retrospective chart review	Aesthetic correction	BONT‐A, HA filler (Belotero Balance), CaHA filler (Radiesse)	USA	n/s, aesthetic treatment patients, face and neck	No serious adverse events in 101 subjects, confirming safe co‐treatment
Friedmann et al., 2014 [72]	Case series	Aging face	IPL, PLLA (Sculptra)	USA	Not specified, not specified, not specified	Safe, effective facial rejuvenation with minimal adverse events in 90 patients
Hart et al., 2015 [71]	Case series	Aging of the face, neck, and décolletage	PLLA (Sculptra)	USA	n/s, n/a, n/s	Efficiently improved face, neck, and décolletage rejuvenation with reduced downtime in one session
Jeon et al., 2018 [100]	Prospective, evaluator‐blinded study	Horizontal neck lines	BONT‐A (IncobotulinumtoxinA), HA filler (Belotero), CaHA filler (Radiesse)	South Korea	24–50 years, 100% female, Asian	Significantly reduced neck wrinkle length by 59% in 6 months
Jerdan & Fabi, 2016 [73]	Expert opinion	Aging face	PLLA (Sculptra), IPL, NIR	USA	n/a, n/a, not specified	Effectively tightened off‐face areas like neck, chest, and thighs, showing significant improvement in skin laxity and wrinkles
Kwon et al., 2018 [79]	Randomized interventional prospective study	Facial laxity	TPIG	South Korea	39–69 years, 100% female, Fitzpatrick skin type III and IV	Histologic analysis confirmed increased dermal collagen fibers post‐treatment, indicating enhanced skin tightening and lifting efficacy
Kwon et al., 2018 [79]	Prospective, evaluator‐blinded study	Facial laxity	MPRF (Thermage CPT System)	South Korea	39–69 years, 100% female, Fitzpatrick skin type III and IV	90% of patients showing moderate to marked improvement in facial skin laxity after 20 weeks
Park et al., 2020 [101]	Prospective, evaluator‐blinded study	Periocular rejuvenation	HA filler (Belotero Balance, Belotero Soft), BONT‐A (IncobotulinumtoxinA)	South Korea	24–58 years, 100% female, Asian	Improved eyebrow height by 3.9 mm, showing significant periocular rejuvenation in 12 weeks
Park et al., 2023 [80]	Retrospective	Enlarged facial pores	BONT‐A (IncobotulinumtoxinA)	South Korea	36.9 ± 5.5 years, 95% female, Asian	Reduced facial pore count by 62% in 6 months, showing sustained improvement without rebound
Ramirez & Puah, 2021 [102]	Prospective, single‐arm	Brachial skin laxity	CaHA filler (Radiesse)	Singapore	35–65 years, 100% female, 25% Asian, 75% Caucasian	Improved brachial skin laxity, increasing skin firmness by 16% in 24 weeks, with high satisfaction
Salomao et al., 2016 [76]	Case report	Facial photoageing	Erbium‐YAG laser, radiofrequency microneedle	Brazil	35–65 years, 100% female, n/s	Significantly improved skin laxity and wrinkles in 40 days
Vanaman et al., 2016 [103]	Case study and expert opinion	Neck rejuvenation	IPL, QS 532694755 nm, CO_2_ fractionated laser, deoxycholate	USA	n/s, n/a, n/s	Improved neck skin laxity by 2 grades in 85% of patients, with minimal downtime
Woodward et al., 2014 [104]	Retrospective	Face and neck laxity	Fractional CO_2_ laser	USA	n/s, not specified, n/s	Combining MFU‐V with fractional CO₂ laser improved skin laxity in 78% of patients, with an 80% satisfaction rate post‐treatment
Wu et al., 2016 [105]	Histological	Aesthetic collagen loss	PMMA filler (Bellafill)	USA	n/s, 100% female, n/s	Combining MFU‐V with PMMA‐collagen filler showed no histological changes in PMMA, confirming safe co‐treatment without adverse effects
Yusova & Stepanov, 2020 [81]	Randomized, comparative study	Aging face	PRP	Russia	40–50 years, 94.29% female, n/s	Improved skin quality, increasing dermal thickness by 19.03% compared to 10.46% with MFU‐V alone in 6 months
Yutskovskaya et al., 2020 [52]	Randomized, split‐face comparative	Skin laxity	CaHA filler (Radiesse)	Russia	35–45 years, 100% female, not specified	Improved marionette lines, jawline contour, and neck scores by 27%, 14%, and 19% in 15 months
Zaleski‐Larsen et al., 2016 [106]	Expert opinion	Acne scars	Fractional CO_2_ laser, microneedling	USA	n/s, n/a, n/s	Synergistic improvement in acne scars, with enhanced texture and skin tightening

Abbreviations: n/a, not applicable; n/s, not stated.

## 7 Adverse Events

The safety profile of MFU‐V has been well‐established across multiple studies and at various anatomies, generally showing only mild and transient adverse events [18]. The most common adverse events include erythema, edema, and welts, which typically appear shortly after treatment and resolve within a few days or weeks. A 52‐patient clinical study by Harris and Sundaram investigated the safety of MFU‐V in Fitzpatrick skin types III to VI, historically a patient demographic that is considered higher risk of adverse events following EBD treatments, and reported only three adverse events (one moderate and two mild) that were self‐limiting and included prolonger erythema with mild scabbing (moderate) and mild edema and welts (mild) [82]. The most frequently reported adverse event in the majority of studies was mild to moderate pain. Pain levels varied based on the treatment area and depth of penetration, with most studies noting that discomfort could be managed with topical analgesics or oral pain medicine [83, 84, 85]. Rare but notable adverse events include burns, scabbing, and prolonged erythema, but have been almost entirely linked to improper device use, such as inadequate coupling between the transducers and patient skin [86]. Overall, proper training and device usage are critical for mitigating moderate to severe adverse events. At the same time, pain management strategies can minimize the most frequent complications associated with MFU‐V treatment.

## 8 Limitations

The current narrative review has several limitations. It does not represent a meta‐analysis or a systematic review but rather a narrative review of scientific and clinical insights from existing literature selected through keyword‐driven searches from various academic sources. The review examines the mechanism of action and the supporting evidence for soft tissue regeneration through MFU‐V without offering specific clinical practices or guidelines. Future meta‐analyses and systematic reviews into the efficacy of MFU‐V are warranted, as are systematic reviews on adverse events. Despite these constraints, the review provides a comprehensive overview, starting from the basic properties of MFU‐V to its role in enhancing soft tissue's structural and functional recovery.

## 9 Conclusions

The presented evidence indicates that MFU‐V can induce significant changes in skin biomechanics, firmness, and elasticity, suggesting its potential for regenerative effects. While studies vary in design, including in vivo and in vitro investigations, animal models, and cadaveric tissue analyses, they provide foundational insights into MFU‐V's mechanism of action. Existing literature outlines the generation of TCPs in the dermis, subcutis, and SMAS. These points induce the denaturation of collagen and supraphysiological tissue heating, initiating a biological process that leads to the synthesis of new collagen and elastin. The transient inflammation related to the body's healing process results in enhanced fibroblast activity and subsequent ECM protein secretion, improving the structural integrity and elasticity of the skin.

While the evidence is promising, there is a need for more quantitative basic research and randomized controlled trials with standardized methodologies and objective outcome measures to elucidate regenerative pathways and capabilities further. Additionally, the current research predominantly focuses on female subjects and specific ethnic groups, limiting the generalizability of the findings, with future research needing to diversify patient demographics to validate MFU's efficacy across different populations.

MFU‐V has also shown promise in combination with other treatments, such CaHA and HA fillers, potentially enhancing outcomes through a synergistic approach. However, more rigorous studies are required to fully understand the extent and durability of these combined effects.

In summary, MFU‐V operates through mechanisms involving TCPs that induce collagen and elastin fiber regeneration, resulting in noticeable improvements in skin structure, firmness, and elasticity. These changes are associated with enhanced skin quality and a reduction in skin laxity, contributing to a more rejuvenated appearance. While MFU‐V demonstrates a capacity to remodel the ECM and improve skin biomechanics, further research is needed to fully elucidate the impact and optimal use within the field of regenerative aesthetics. Future studies should focus on expanding patient diversity and employing objective assessments to deepen our understanding of MFU‐V's role in aesthetic medicine and its potential applications, particularly when used in combination with other treatments like CaHA and HA fillers.

## Author Contributions

All authors made equally significant contributions to the concept, design, and execution as this consensus method manuscript.

## Disclosure

Drs. Akers, Jackson, and McCarthy are employed by Merz Aesthetics (Raleigh, NC).

## Ethics Statement

No human participants or animals were involved in the manuscript. This manuscript represents original work conducted with commitment to ethical research practices.

## Conflicts of Interest

Dr. Vachiramon is a consultant and speaker for Merz Aesthetics, Beiersdorf, and L’Oreal. Dr. Pavicic is a consultant and speaker for Merz Aesthetics and Advanced Aesthetic Technologies, and an investigator for Merz Aesthetics, AbbVie, AAT, LG, and Croma. Dr. Casabona consults for Merz Aesthetics. Dr. Green speaks, advises, and conducts clinical trials for Allergan Aesthetics, Croma‐Pharma GmbH, Crown Pharmaceuticals, Inc., Cutera, Galderma, L’Oreal USA, Merz Aesthetics, Revance Therapeutics, Inc., Revelle Aesthetics, Silk Medical Aesthetics, Inc., and SkinBetter Science. Dr. Levine consults and speaks for Allergan and BTL, advises Galderma and Merz Aesthetics, and speaks for RVL. Dr. Park consults for Merz Pharmaceuticals, Allergan, and LG Chem. Dr. Spada is a consultant and speaker for Merz Aesthetics. Dr. Muniz is a medical consultant and speaker for Merz Aesthetics. Drs. Akers, Jackson, and McCarthy are employees of Merz Aesthetics.

## Data Availability

Data sharing not applicable to this article as no datasets were generated or analysed during the current study.
